# Liver Transplantation in 3 Cholestatic Infants With History of COVID Exposure

**DOI:** 10.1177/10935266251325335

**Published:** 2025-03-28

**Authors:** Shruti Sakhuja, Kalyani R. Patel, Matthew Goss, Flor M. Munoz, Garrett Wortham, Megan Crawford, John A. Goss, Nhu Thao Galvan

**Affiliations:** 1 Department of Pediatrics, Division of Gastroenterology, Hepatology, and Nutrition, Baylor College of Medicine and Texas Children’s Hospital, Houston, TX, USA; 2 Department of Pathology, Baylor College of Medicine and Texas Children’s Hospital, Houston, TX, USA; 3 McGovern Medical School, University of Texas Health Science Center at Houston, Houston, TX, USA; 4 Department of Pediatrics, Division of Infectious Diseases, Baylor College of Medicine and Texas Children’s Hospital, Houston, TX, USA; 5 Baylor College of Medicine, Houston, TX, USA; 6 Department of Surgery, Division of Abdominal Transplantation, Baylor College of Medicine, Houston, TX, USA

**Keywords:** SARS-CoV-2, cholestasis, cholangiopathy, pregnancy, neonates, transplant

## Abstract

The COVID-19 pandemic presents several challenges during pregnancy including thromboembolic complications, direct placental infection, transplacental transmission, and systemic hyperinflammatory state. The liver is the second most commonly affected organ in SARS-CoV-2 infection after the lungs. Mechanisms of liver injury in COVID-19 patients can include: direct viral cytopathic effect, worsening of underlying liver disease, cytokine storm, hypoxic ischemic injury, and cholangiopathy leading to persistent marked cholestasis. Here we describe 3 infants at Texas Children’s Hospital with perinatal SARS-CoV-2 exposure with persistent cholestasis and histologic evidence of extrahepatic biliary obstruction suggesting underlying biliary atresia (BA) with some atypical features possibly exacerbated by SARS-CoV-2 infection. All 3 patients described in this case series developed liver failure in the setting of low GGT cholestasis, and all 3 required liver transplantation within the first year of life. Though post-COVID cholangiopathy is described in adults in the literature, none of the infants in our series had moderate or severe COVID infection but still progressed to advanced liver disease. Instead it is very likely that the patients in our series had underlying BA with some atypical features, with the commonality of having been exposed perinatally to SARS-CoV-2 Though further studies are needed to determine causality, our case series raises the question of if the timing of exposure/infection plays a role in prognosis.

## Background

The pandemic presents a unique set of challenges in pregnancy.^1^ COVID-19 is associated with a higher rate of thromboembolic complications, with 1 study by Knight et al^2^ suggesting a thrombotic event rate as high as 31% in critically ill patients. This rate of thromboembolic complications varies based on strains of SARS-CoV-2 since the inception of the pandemic.^3^ Pregnant women with COVID-19 have synergistic risk factors for thrombosis.^4^ Thrombosis in turn can potentially lead to ischemic injury of the hepatobiliary system by way of intrahepatic microangiopathy, thereby causing cholestatic liver disease.^5,6^

Reported mechanisms of liver injury in COVID-19 patients include: direct viral cytopathic effect, drug-induced hepatotoxicity, worsening of underlying liver disease, hyperinflammatory cytokine storm, hypoxic ischemic injury, and cholangiopathy.^7,8^ Post-COVID-19 cholangiopathy is thought to be a variant of secondary sclerosing cholangitis in critically ill patients (SSC-CIP) but with unique histopathologic features such as severe cholangiocyte injury and intrahepatic microangiopathy.^9^ Patients with this manifestation have marked cholestasis with ongoing jaundice that persists long after other organs have recovered from infection, and some may require liver transplantation.^10^ To date only a handful of liver transplants have been reported for post-COVID-19 cholangiopathy, all in ill adults after severe COVID-19 and none in children.^6,10,11^

Our series describes 3 infants with perinatal SARS-CoV-2 exposure with a unique combination of low gamma glutamyl transferase (GGT) cholestasis paired with extrahepatic biliary obstruction consistent with biliary atresia (BA), leading to liver failure and requiring an orthotopic liver transplant (OLT). Given their unusual presentation, we reviewed their clinical records in detail.

## Methods

We conducted a medical record review of 3 infants with perinatal SARS-CoV-2 exposure who required liver transplantation for cholestatic liver disease at Texas Children’s Hospital (TCH) from March 2020 to September 2021. IRB approval was obtained for this study (H-22045).

Diagnosis of SARS-CoV-2 infection was made in the 2 mothers and 1 patient by documentation of a positive SARS-CoV-2 polymerase chain reaction (PCR) result or positive SARS-CoV-2 spike protein antibodies. The perinatal period was defined as starting near the end of the first trimester of pregnancy and leading up to 1 month after birth. Cholestasis was defined by marked reduction in bile secretion and flow, as evidenced by elevation in direct bilirubin. Patients were diagnosed with cholangiopathy due to evidence of extrahepatic biliary obstruction, marked cholestasis, severe cholangiocyte injury, and intrahepatic microangiopathy.^12^ Descriptive statistics were used for maternal and patient demographic characteristics. Statistical analyses were performed in Stata v 15 (StataCorp LLC, College Station, TX).

## Results

Further exploration of the 3 patients’ birth history and medical history led to a COVID-19 association during 2020. The 3 patients are from Southern regions that were hardest hit by the COVID-19 pandemic during that time. All had exposure to the virus during the perinatal period, either through infection after birth or presumably indirectly through maternal infection as evidenced by history and positive SARS-CoV-2 spike protein antibodies. These 3 patients presented with signs and symptoms of liver failure during infancy. Their demographic characteristics and clinical findings are summarized in Table 1.

**Table 1.** Demographic, Clinical, and Imaging Findings During the Course of the Illness.

	Patient # 1	Patient # 2	Patient # 3
Mother			
Age at delivery (y)	26	37	29
Gravida	G5P3113	G5P4	G3P2
COVID-19 infection	First trimester	None	At delivery
Severity	Mild	Not applicable	Asymptomatic
Baby at birth			
Gestation (wk)	37	39	37
Gender	M	F	F
Birth weight (g)	3310	Unknown	Unknown
Birth length (cm)	50	Unknown	Unknown
Otherwise healthy?	Yes	HbSS (Sickle cell ds)	Yes
First increased d-bil	1 wk (1.5 mg/dL)	2 mo	3 mo
First clinical presentation			
Age	7 mo	2 mo	3 mo
Liver Tests:			
AST (U/L)	492 (H)	2911 (H)	836 (H)
ALT (U/L)	305 (H)	1528 (H)	207 (H)
ALP (U/L)	1549 (H)	490 (H)	2865 (H)
GGT (U/L)	44 (N)	99 (N)	101 (N)
T-bil (mg/dL)	23.8 (H)	29.5 (H)	28.6 (H)
D-bil (mg/dL)	14.4 (H)	22.1(H)	14 (H)
INR	1.9 (H)	1.4 (H)	6.2 (H)
Covid tests:			
RT-PCR	Negative	Positive	Negative
Antibodies	Reactive	Reactive	Reactive
Other infection?	No	No	CMV PCR positive
Imaging:			
Liver	Cirrhosis	Hepatomegaly	Cirrhosis
Splenomegaly	Yes	Yes	Yes
Varices	No	No	No
GB	Normal	Absent	Small with thick wall
Triangular cord sign	Not seen	Not seen	Not seen
Bile duct dilatation	No	No	No
HIDA scan	Not doe	Homogenous uptake, no drainage	Not done
IOC	Not done	Not done	Not done
Liver biopsy			
Age	7 mo	2 mo	3 mo
Diagnosis	Distal biliary obstruction, stage 3 fibrosis	Idiopathic neonatal giant cell hepatitis.	Obstructive cholangiopathy
Cholestasis panel	No	VOUS at PKHD1 gene	No
Transplant age	9 mo	6 mo	3.5 mo

### Case 1

**Figure 1.**
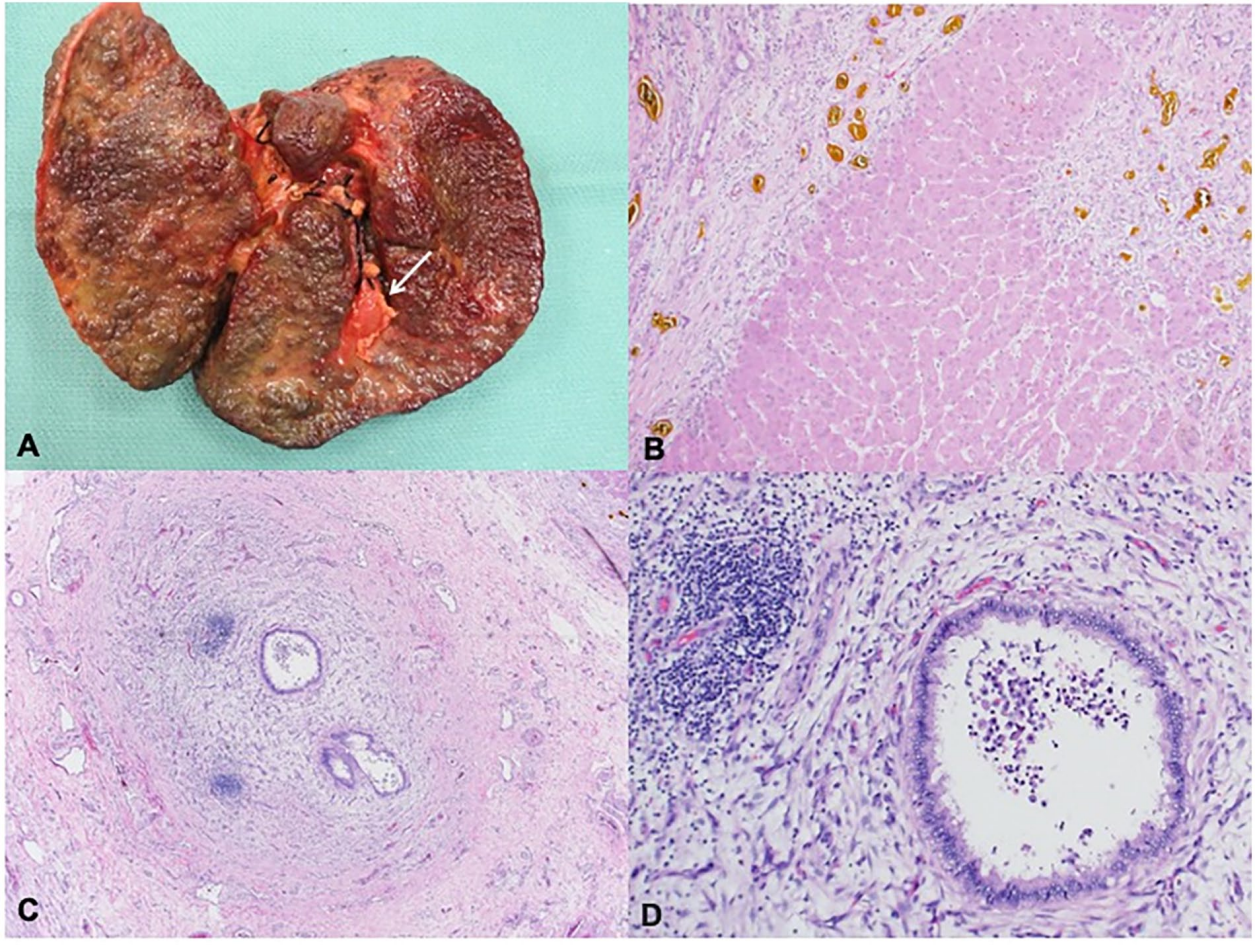
Explant pathology for Case 1. (A) Liver explant at 9 months of age shows a diffusely nodular, cirrhotic liver with a hypoplastic gallbladder. (B) Parenchyma showed micro and macronodular cirrhosis with broad fibrous bands, extensive ductular reaction and bile plugs (H&E, 100×). (C and D) Hilar bile ducts showed attenuated lumen, reactive epithelium with periductal fibroplasia and chronic inflammation and focal intraluminal neutrophils (H&E, 40× and 200× respectively).

The first patient is a male born at 37 weeks’ gestation to a 26-year-old gravida 5, para 3 (G5P3113) mother who was diagnosed with SARS-CoV-2 infection by PCR testing when she had cold-like symptoms and fever during her first trimester of pregnancy in early spring 2020. At 1 week of life his conjugated bilirubin was 1.5 mg/dL and peaked at 2.6 mg/dL at 11 days. He had a febrile illness at 2 weeks of life for which he presented to a local emergency room but was not tested for SARS-CoV-2. He was then lost to follow up with his pediatrician due to fear of exposure to SARS-CoV-2 until about 7 months of life, when he developed worsening jaundice and direct hyperbilirubinemia, prompting admission and evaluation. His pertinent laboratory results at this time showed elevated AST 492 U/L, ALT 305 U/L, ALP 1549 U/L, total bilirubin 23.8 mg/dL, direct bilirubin 14.4 mg/dL, and INR 1.9 with normal GGT 44 U/L. He had positive SARS-CoV-2 spike protein IgM antibodies, but negative PCR. Of note his urine bile acid test was positive for 5 beta reductase deficiency, and he was preliminarily diagnosed with bile acid synthesis defect. An ultrasound (US) abdomen with doppler showed enlarged cirrhotic liver with sequelae of portal hypertension such as splenomegaly and to-and-fro flow within the portal vein. He had evidence of splenorenal shunt and elevated resistive indices of the hepatic arteries, but no evidence of varices. A liver biopsy at 7 months of age showed distal biliary obstruction with stage 3 fibrosis (material not available for review from outside hospital). He received orthotopic liver transplantation with Roux-en-Y hepaticojejunostomy at 9 months of life. Explant showed biliary cirrhosis (Figure 1(a) and (b)) with features of obstructive cholangiopathy and atretic bile ducts, consistent with BA (Figure 1(c) and (d)). Gallbladder was hypoplastic showing mural fibroplasia. Hilar bile ducts were attenuated with periductal fibroplasia and chronic inflammation. Giant cell transformation, characteristic of bile acid synthesis defect, was focal and minimal. Though his initial urine bile acid test was positive for 5 beta reductase deficiency, genetic testing confirmed no evidence of bile acid synthesis defect. His post-transplant course was complicated by an episode of severe acute cellular rejection, treated with a steroid pulse to excellent response.

### Case 2

**Figure 2.**
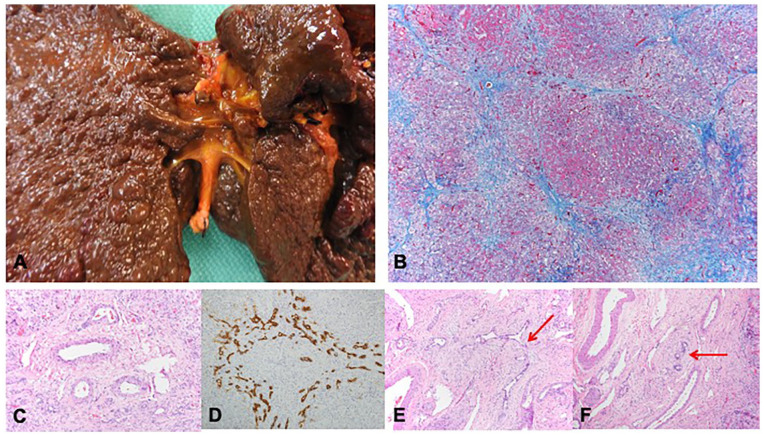
Explant pathology for Case 2. (A and B) Explant shows absent gallbladder with diffuse micronodular biiary cirrhosis (B: Trichrome, 100×). (C) Intrahepatic portal tract showing absent of bile duct (H&E, 200×). (D) Intrahepatic portal tract showing a preserved bile dict with marked ductular reaction by immunostain for cytokeratin 7 (200×). (E) Intrahepatic portal tract showing a sclerosing bile duct-like lesion (H&E, 200×). (F) Hilar region showing large arteries and veins with attenuated bile ducts and periductal fibroplasia (H&E, 200×).

Our second patient is a former full-term female with sickle cell (HbSS) disease. At around 1 month of life in the summer of 2020, the infant presented to an outside facility with jaundice and scleral icterus. She was tested for several hepatitis-associated viruses and was found to have SARS-CoV-2 infection with positive PCR and total spike protein antibodies, as well as significant elevation of her AST 2911 U/L, ALT 1528 U/L, ALP 490 U/L, total bilirubin 29.5 mg/dL, direct bilirubin 22.1 mg/dL, and INR 1.4. Her GGT was 99 U/L, which was normal for her age. Maternal grandmother had also tested positive for SARS-CoV-2 by PCR and mom had symptoms of COVID-19 but was not tested. Initial RUQ US showed hepatomegaly with slight echogenic appearance of the liver, and no visualization of the gallbladder. Subsequent hepatobiliary iminodiacetic acid (HIDA) scan showed homogenous radiotracer uptake in the liver without associated gallbladder or intestinal activity. She underwent liver biopsy which was reported as idiopathic neonatal hepatitis with cirrhosis (material not available for review from outside hospital). Her hepatitis was presumed to be related to SARS-CoV-2 as this is a less common, but possible, presentation of BA, and she was discharged home in stable condition with subsequent down-trending of her aminotransferases and GGT. However, her cholestasis continued to worsen, so she was admitted to TCH and ultimately received orthotopic liver transplantation (whole organ, Roux-en-Y hepaticojejunostomy end-to-side with stent placement) at 6 months of life. Explant pathology revealed diagnostic features of BA, including an absent gallbladder, biliary cirrhosis, and complete atresia of the extrahepatic biliary tract (Figure 2(a)-(f)). Her post-transplant course was complicated by hypertension and 1 episode of mild acute cellular rejection that was successfully treated with pulse steroids. Genetic testing for causes of cholestasis was negative.

### Case 3

**Figure 3.**
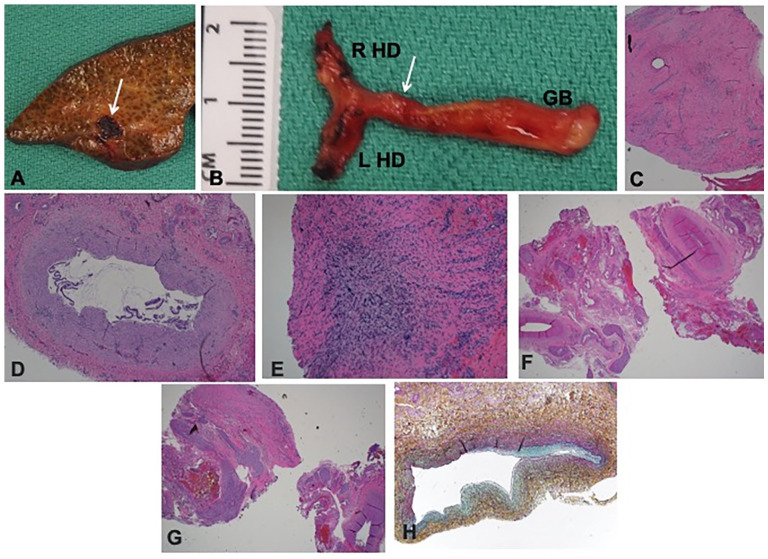
Explant pathology for Case 3. (A) Explant shows marked parenchymal fibrosis with bile lakes (arrow). (B) Gallbladder (GB) was dissected along with right and left hepatic ducts (R HD and L HD). Arrow indicates the site at which the duct margin was sampled. (C) Sampled duct margin showed no lumen by both macro and microscopic examination (H&E, 40×). (D) Gallbladder appeared hypoplastic with an identifiable lumen in the fundus and body (H&E, 40×). (E) However, the cystic duct was not probe patent and showed complete luminal atresia by microscopic examination (H&E, 40×). (F and G) Right and left hepatic ducts showed fibrovascular tissue only without any identifiable lumen (H&E, 40×). (H) Portal vein shows variable luminal occlusion by medial and intimal fibromyxoid hyperplasia (Movat pentachrome stain, 200×).

This was a full-term female baby born at home to a gravida 3, para 2 (G3P2) mother who had no prenatal care and tested positive for SARS-CoV-2 by PCR on admission to the hospital shortly after delivery in spring 2020. The mother had no symptoms of COVID-19. The patient was subsequently lost to follow up, but presented to their local emergency room at 3 months of life with bleeding from her umbilicus and jaundice. She was found to have the following lab abnormalities at that time: AST 836 U/L, ALT 207 U/L, ALP 2865 U/L, total bilirubin 28.6 mg/dL, direct bilirubin >14 mg/dL, and INR 6.2. GGT was 101 U/L, which was normal for age. Her CMV blood PCR was found to be positive during her work up for neonatal cholestasis, and SARS-CoV-2 spike protein IgM antibody was also positive but her PCR negative. Her RUQ US showed mild ascites, homogenous liver without focal parenchymal abnormality, and normal hepatic venous and arterial flow, suggestive of acute liver injury. Liver biopsy showed marked ductular proliferation and fibrosis, as well as severe cholestasis, which can be seen in both obstructive cholangiopathy and BA. She underwent orthotopic liver transplantation at 3 months of life, with her post-operative course complicated by difficulty with extubation due to respiratory failure, as well as difficulty advancing feeds. Her liver explant pathology showed distal biliary obstruction with diffuse biliary cirrhosis, marked lobular cholestasis, complete fibrous occlusion of the right and left hepatic duct, common bile duct, and cystic duct, suggesting a BA phenotype (Figure 3(a)-(h)). Again, genetic testing for causes of cholestasis was negative.

## Discussion

Here we present hepatic and extrahepatic findings that are seen in BA in 3 neonates with perinatal exposure to SARS-CoV-2. None had severe COVID themselves and did not require medical care or hospitalization for respiratory problems. All presented with progressive, low GGT cholestasis in infancy leading to liver failure and OLT within 1 year of age. All 3 were otherwise healthy full-term babies with unremarkable prenatal courses. One had an underlying condition causing neonatal cholestasis (sickle cell disease) and 1 showed concurrent CMV infection that can cause cholangiopathy. Explanted livers showed biliary cirrhosis and extrahepatic findings mimicking cholangiopathy, but more consistent with BA (Table 2). In fact, the clinical definition of BA is biliary cirrhosis with atretic bile ducts, which was true of all 3 of our patients.^13^ Furthermore, these explants lacked the hallmark features of pure COVID-19 cholangiopathy—beading of the intrahepatic bile duct with a normal extrahepatic biliary tree.^6^ Viral PCR studies for Adenovirus, Parvovirus, EBV, and CMV were negative for all 3 liver explants.

**Table 2.** Comparative Explant Pathology.

Pathologic Finding	Patient # 1	Patient # 2	Patient # 3	Ref
Extrahepatic				
CBD	Complete atresia	Absent	Complete atresia	—
Gallbladder	Hypoplasia	Absent	Hypoplasia	—
Cystic duct	Complete atresia	Absent	Complete atresia	—
CHD	Hypoplasia, periductal fibroplasia	Complete atresia	Complete atresia	—
Rt HD	Hypoplasia, periductal fibroplasia	Complete atresia	Complete atresia	—
Lt HD	Hypoplasia, periductal fibroplasia	Complete atresia	Complete atresia	—
Hilar bile ducts	Hypoplasia, periductal fibroplasia	Hypoplasia, periductal fibroplasia	Hypoplasia, periductal fibroplasia	Durazo Tr proceedings 2021
Portal vein	Intimal fibromyxoid hyperplasia	Normal	Medial and Intimal fibromyxoid hyperplasia	—
Hepatic artery	Normal	Normal	Normal	Durazo
Intrahepatic				
Portal fibrosis	Stage 4	Stage 4	Stage 4	Tafreshi, Roth
Ductular reaction	Marked	Marked	Marked	Tafreshi, Roth, Lagana
Duct loss	Yes	Yes	Yes	Roth
Cholangiocyte injury	No	Yes	No	Roth
Bile duct plugs	Yes, diffuse	Yes, few	Yes, diffuse	—
Bile lakes	Yes	No	Yes	Durazo, Tafreshi
Microabscesses	No	No	No	Durazo
SC-like lesions	No	Yes	No	Durazo
Cholestasis	Marked	Marked	Marked	Tafreshi, Lagana
Steatosis	No	No	No	Lagana
Acute liver injury	No	No	No	Lagana
Reg nodules	Yes	Yes	Yes	—
Giant cell change	Focal, minimal	Diffuse	Diffuse	—
Sinusoidal thrombi	No	No	No	Lagana
OPV	Yes	No	Yes	Durazo, Roth, Lagana
Microarteriopathy	No	No	No	Durazo, Roth, Lagana
CV thrombi	No	No	No	Roth, Lagana
Tissue RT-PCR for Sars-Cov-2	Negative	Negative	Negative	—

Abbreviations: CBD, common bile duct; CHD, common hepatic duct; CV, central vein; HA, hepatic artery; Lt HD, left hepatic duct; OPV, obliterative portal venopathy; PV, portal vein; Rt HD, right hepatic duct.

It is worth noting in these cases is that although BA is most commonly associated with a high GGT, it is possible for patients to present with a normal or low GGT as is the most likely case in our series.^14^ A study conducted in Australia with 113 BA infants showed that 12.3% of the cohort had normal GGT at presentation, and outcomes were poorer in this particular cohort compared to the group with elevated GGT.^14^ Therefore the possibility of BA being the etiology of the liver disease in the 3 patients described in this case series cannot be excluded based on their normal GGT levels alone. Transient neonatal cholestasis has been described in patients with sickle cell disease,^12^ however none required LT within 1 year of life. It is possible that HbSS phenotype played a role in the cholestasis seen in patient 2, or may have altered the course of cholangiopathy, but HbSS alone would not have led to obstructive cholangiopathy or LT. On the other hand, patient 3 had CMV viremia at the time of presentation which is known to cause progressive cholestasis leading to LT. In patients with double viral infections, both of which potentially cause cholangiopathy, it is difficult to isolate the role played by each. It is possible that the viral cytopathic effect was synergistic or 1 virus was a mere bystander. Tissue PCR for CMV was negative in patient 3, and notably, there is no standardized way of isolating SARS-CoV-2 from liver tissue at this time.

**Figure 4.**
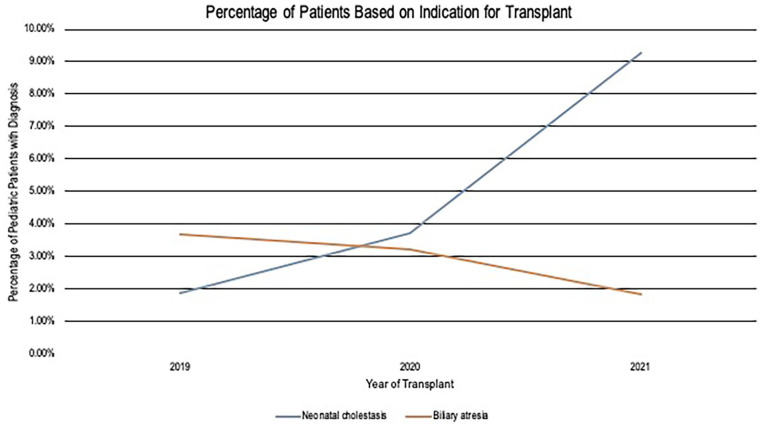
Percentage of patients by indication for transplant.

All previous reports of post-COVID cholangiopathy include adult patients with moderate to severe COVID, some requiring a prolonged intensive care unit stay.^15^ Studies showing elevated liver biochemical tests including direct bilirubin and alkaline phosphatase are also largely conducted in hospitalized patients and show that cholestasis is overall rare in COVID patients (about 1%) but, when present, confers a poor prognosis.^16^ There are few reports of jaundice and acute cholestatic hepatitis in children and adolescents with mild COVID-19^17,18^ and some in adults.^19,20^ Most show normalization of direct bilirubin in 2–3 weeks. In a study of 16 640 infants admitted to the Neonatology department in southwest China between January 2019 and August 2020, the percentage of patients who suffered from respiratory diseases, neonatal encephalopathy, and infectious diseases decreased, while the percentage of pathological jaundice-related conditions and diseases of the gastrointestinal system increased.^21^ Our examination of the SRTR database using Stata v 15 from 2019 to 2021 showed a twofold increase in pediatric OLT for cholestasis that were not due to BA (Figure 4). While this does not suggest a causal relationship, it does warrant closer examination at the potential direct or indirect association between perinatal SARS-CoV-2 and neonatal cholangiopathy. Additionally, our patients progressed to early advanced liver disease despite only mild COVID infection, raising the question of if the timing of exposure/infection during the perinatal period may play a more significant direct or indirect role in liver pathology than previously understood.

The pathogenic mechanisms of post-COVID cholangiopathy are varied and include direct viral cytopathic effect, worsening of underlying liver disease, hyperinflammatory cytokine storm, and hypoxic ischemic injury. Cholangiocytes express ACE-2 receptors at levels similar to the lungs and greater than hepatocytes, which may increase their susceptibility to direct viral damage.^6^ Two of the 3 infants were never RT-PCR positive suggesting that they were not acutely infected with SARS-CoV-2, and the interval between PCR positivity and LT for the third patient was 5 months. All 3 patients had positive IgM spike antibodies which suggests the development of immunity to SARS-CoV-2 due to previous exposure. Although the explants showed inflamed bile ducts with reactive cholangiocytes showing cytologic features similar to those described in previous reports, these changes are non-specific and can be induced by other factors such as bile toxicity and hypoxia. Bile ducts are at higher risk of ischemic injury during a hypoxic state than hepatocytes due to lack of dual arterial supply. We did not identify macro- or microarteriopathy in our series, making hypoxic injury of the bile ducts less likely. However, our series is limited by the timing of tissue assessment (i.e., explants) that is far out from the postulated SARS-CoV-2 insult. It is possible that arteriopathy resolves over time making the residual bile duct injury prominent at the time of explant.^10,11^ Lagana et al^5^ additionally describes hepatobiliary injury as a result of ischemia from microvascular coagulopathy, with 15% of the explant pathology they reviewed showing sinusoidal microthrombi.^6^ ACE-2 receptors are also expressed diffusely in endothelial cells of small and large blood vessels possibly playing a role in widespread coagulation leading to morbidity and mortality in patients with COVID-19.^11^ And as described earlier in this series, pregnant women are at even higher risk of coagulation given their baseline hypercoagulable state, which in turn may further increase the risk of fetal vascular malperfusion.^2,4,7^ Scattered sinusoidal platelet aggregates were seen in our explants without intravascular thrombi. In addition to bile duct ischemia, a hyperinflammatory state induced by infection can lead to toxic injury, cholangiocyte necrosis, and development of sclerosing cholangitis and cholangiopathy.^10^ A hyperinflammatory state in pregnant women may lead to placental malperfusion and have detrimental impacts on the development of the fetus that are starting to be uncovered.^4^ Our patients never presented with clinical signs and symptoms of a hyperinflammatory state themselves. However, a recent study conducted by Wastnedge et al^4^ showed increased fibrin deposition and villous trophoblast necrosis, as well as histiocytic intervillositis, in 100% of the placentas of pregnant women with COVID-19. SARS-CoV-2 placentitis is believed to cause severe placental destruction resulting in placental malperfusion and insufficiency which in turn can cause neonatal death due to hypoxic-ischemic injury.

Another proposed mechanism of cholangiopathy in patients with COVID-19 involves destruction of the protective mechanisms of cholangiocytes, and therefore toxic injury from buildup of bile acids,^10^ which is a possibility in our patients. Lastly, patients with pre-existing liver diseases tend to show increased incidence of SARS-CoV-2-related hepatic injury. The primary etiology of liver disease in these patients is most consistent with BA, with SARS-CoV-2 infection as a potential complicating factor contributing to early disease progression.

There are striking similarities between the presumed pathogenesis of COVID cholangiopathy and BA. Perinatal BA is thought to be due to progressive inflammation and fibro-obliteration caused by a perinatal insult, whether infectious, toxic, vascular, or immune-mediated.^22^ This potentially triggers an inflammatory response in the bile ducts leading to their apoptosis, fibrosis, and extrahepatic obstruction.^22^

At this time it is uncertain how much of the liver injury evidenced in our patients was due to direct cytopathic effect of the SARS-CoV-2 virus itself, a microvascular insult secondary to COVID-19, ischemia of cholangiocytes, or worsening of an underlying liver disease such as BA. Given the relatively mild severity of SARS-CoV-2 infection as well as the lack of definitive microangiopathy seen on histology, it is challenging to assign causality to SARS-CoV-2 alone for the patients in this series. It is highly likely that the primary etiology of liver disease in these patients was extrahepatic BA further complicated by concurrent SARS-CoV-2 infection. Future studies that can further elucidate this association and also assess the long-term effects of perinatal SARS-CoV-2 exposure on the neonatal biliary system are warranted.

## References

[1] Metz TD Clifton RG Hughes BL , et al; National Institute of Child Health and Human Development Maternal-Fetal Medicine Units (MFMU) Network. Association of SARS-CoV-2 infection with serious maternal morbidity and mortality from obstetric complications. JAMA. 2022;327(8):748-759. 35129581 10.1001/jama.2022.1190 PMC8822445

[2] Knight M Bunch K Vousden N , et al; UK Obstetric Surveillance System SARS-CoV-2 Infection in Pregnancy Collaborative Group. Characteristics and outcomes of pregnant women admitted to hospital with confirmed SARS-CoV-2 infection in UK: national population based cohort study. BMJ. 2020;369:m2107. 10.1136/bmj.m2107 PMC7277610 32513659

[3] Law N Chan J Kelly C , et al. Incidence of pulmonary embolism in COVID-19 infection in the ED: ancestral, Delta, Omicron variants and vaccines. Emerg Radiol. 2022;29:625-629. 35446000 10.1007/s10140-022-02039-z PMC9022402

[4] Wastnedge EAN Reynolds RM van Boeckel SR , et al. Pregnancy and COVID-19. Physiol Rev. 2021;101(1):303-318. 32969772 10.1152/physrev.00024.2020 PMC7686875

[5] Lagana SM Kudose S Iuga AC , et al. Hepatic pathology in patients dying of COVID-19: a series of 40 cases including clinical, histologic, and virologic data. Mod Pathol. 2020;33:2147-2155. 32792598 10.1038/s41379-020-00649-x PMC7424245

[6] Faruqui S Okoli FC Olsen SK , et al. Cholangiopathy after severe COVID-19: clinical features and prognostic implications. Am J Gastroenterol. 2021;116(7):1414-1425. 33993134 10.14309/ajg.0000000000001264

[7] Ekpanyapong S Bunchorntavakul C Reddy KR. COVID-19 and the liver: lessons learnt from the EAST and the WEST, a year later. J Viral Hepat. 2022;29(1):4-20. 34352133 10.1111/jvh.13590 PMC8446947

[8] Sirinawasatien A Chantarojanasiri T Ekpanyapong S Tivatunsakul N Luvira V. Coronavirus disease 2019 gastrointestinal and liver manifestations in adults: a review. JGH Open. 2021;5(11):1257-1265. 34816011 10.1002/jgh3.12671 PMC8593773

[9] Roth NC Kim A Vitkovski T , et al. Post-COVID-19 cholangiopathy: a novel entity. Am J Gastroenterol. 2021;116(5):1077-1082. 33464757 10.14309/ajg.0000000000001154

[10] Durazo FA Nicholas AA Mahaffey JJ , et al. Post-covid-19 cholangiopathy-a new indication for liver transplantation: a case report. Transplant Proc. 2021;53(4):1132-1137. 33846012 10.1016/j.transproceed.2021.03.007 PMC7953456

[11] Nardo AD Schneeweiss-Gleixner M Bakail M Dixon ED Lax SF Trauner M. Pathophysiological mechanisms of liver injury in COVID-19. Liver Int. 2021;41(1):20-32. 33190346 10.1111/liv.14730 PMC7753756

[12] Allali S de Montalembert M Brousse V , et al. Hepatobiliary complications in children with sickle cell disease: a retrospective review of medical records from 616 patients. J Clin Med. 2019;8(9):1481. 10.3390/jcm8091481 PMC6780325 31540390

[13] Witt M van Wessel DBE de Kleine RHJ Bruggink JLM Hulscher JBF Verkade HJ ; NeSBAR (Netherlands Study group on Biliary Atresia Registry). Prognosis of biliary atresia after 2-year survival with native liver: a nationwide cohort analysis. J Pediatr Gastroenterol Nutr. 2018;67(6):689-694. 30095577 10.1097/MPG.0000000000002130

[14] Shankar S Bolia R Foo HW , et al. Normal gamma glutamyl transferase levels at presentation predict poor outcome in biliary atresia. J Pediatr Gastroenterol Nutr. 2020;70(3):350-355. 31738295 10.1097/MPG.0000000000002563

[15] Bender JM Worman HJ. Jaundice in patients with COVID-19. JGH Open. 2021;5(10):1166-1171. 34622003 10.1002/jgh3.12645 PMC8485400

[16] Da BL Suchman K Roth N , et al; Northwell COVID-19 Research Consortium. Cholestatic liver injury in COVID-19 is a rare and distinct entity and is associated with increased mortality. J Intern Med. 2021;290(2):470-472. 33786906 10.1111/joim.13292 PMC8250628

[17] Perez A Kogan-Liberman D Sheflin-Findling S Raizner A Ahuja KL Ovchinsky N. Presentation of severe acute respiratory syndrome-coronavirus 2 infection as cholestatic jaundice in two healthy adolescents. J Pediatr. 2020;226:278-280. 32710910 10.1016/j.jpeds.2020.07.054 PMC7375964

[18] Pessoa NL Bentes AA de Carvalho AL , et al. Case report: hepatitis in a child infected with SARS-CoV-2 presenting toll-like receptor 7 Gln11Leu single nucleotide polymorphism. Virol J. 2021;18(1):180. 34482844 10.1186/s12985-021-01656-3 PMC8418785

[19] Balaja WR 3rd Jacob S Hamidpour S Masoud A. COVID-19 presenting as acute icteric hepatitis. Cureus. 2021;13(7):e16359. 10.7759/cureus.16359 PMC8360263 34395136

[20] Essa RA Ahmed SK Bapir DH Abubakr CP. Hyperbilirubinemia with mild COVID-19 patient: a case report. Int J Surg Case Rep. 2021;82:105958. 33968603 10.1016/j.ijscr.2021.105958 PMC8091724

[21] Liu W Yang Q Xu ZE , et al. Impact of the COVID-19 pandemic on neonatal admissions in a tertiary children’s hospital in southwest China: an interrupted time-series study. PLoS ONE. 2022;17(1):e0262202. 10.1371/journal.pone.0262202 PMC8758080 35025931

[22] Antala S Taylor SA. Biliary atresia in children: update on disease mechanism, therapies, and patient outcomes. Clin Liver Dis. 2022;26(3):341-354. doi:10.1016/j.cld.2022.03.001 35868678 PMC9309872

